# Management of Posterior Sternoclavicular Joint Dislocation in a Teenager After a Direct Elbow Strike to His Clavicle: A Case Report

**DOI:** 10.7759/cureus.49916

**Published:** 2023-12-04

**Authors:** Mahmut Gorkem Gurcinar, Mete Ozer, Muhammed Yusuf Afacan, Sinan Ustundag

**Affiliations:** 1 Department of Orthopaedics and Traumatology, Istanbul University-Cerrahpaşa, Cerrahpaşa Faculty of Medicine, Istanbul, TUR; 2 Department of Orthopaedics and Traumatology, Istanbul Gelisim University, Istanbul, TUR

**Keywords:** teenager sports injury, dyspnea, direct trauma to clavicle, closed reduction, posterior sternoclavicular joint dislocation

## Abstract

Posterior dislocation of the sternoclavicular joint is a rare orthopedic injury and may result in complications with high mortality due to the location of the joint, accompanied by neurovascular, tracheal, and esophageal injuries. Therefore, an immediate diagnosis and treatment are necessary to prevent complications. In this case, a 13-year-old male patient received an elbow strike to the left clavicle while playing football. The patient presented to the emergency department with complaints of pain, a gap and deformity in the superior and medial part of his sternum, and numbness in his left upper extremity. For this orthopedic emergency, which is difficult to recognize on direct radiographs, a computed tomography was done, which detected a left sternoclavicular joint posterior dislocation. A closed reduction procedure was performed on the patient under sedation in the operating theatre. A serendipity view with the fluoroscopy showed a successful closed reduction. A shoulder arm sling was applied and the patient was followed regularly. In the fourth week, the shoulder arm sling was removed and exercises were started to increase joint movements. In the sixth week, muscle strengthening exercises were started and in the eighth week, a full range of motion was reached with full muscle strength without any deformity. In this case, possible mortal complications were prevented with early intervention in the posterior dislocation of the sternoclavicular joint. This case report shows that with timely rehabilitation, it is possible to achieve full joint range of motion of the shoulder and full muscle strength without deformity or the need for surgery.

## Introduction

The sternoclavicular joint (SCJ) is a sole synovial joint that connects the axial and appendicular skeletons. The component most often damaged in the event of sternoclavicular trauma is the intra-articular disk, also known as the meniscus, which connects to both the anterior and posterior portions of the medial clavicle. Protraction and retraction occurring between the disk and the sternum, and elevation and depression occurring between the clavicle and disk are two compartments in the SCJ. Static and dynamic stability of the SCJ is preserved via the ligaments and muscular attachments [1].

Posterior SCJ injuries are rare. It accounts for less than 5% of shoulder girdle injuries. Posterior dislocations of the SCJ are more dangerous due to their proximity to the mediastinal structures and constitute only 3% of all joint dislocations around the shoulder [1]. Posterior dislocations in children and adolescents are dislocations of the medial physis of the clavicle since ossification of the clavicle continues until 25 years of age [2]. In addition to the rarity of sternoclavicular dislocations, the fact that posterior dislocations have complications such as brachial plexus injury, compression of major vascular structures, respiratory distress due to tracheal compression, hoarseness, dysphagia, and even death increases the importance of these dislocations [3,4]. It is common in athletes involved in sports such as football, American football, and rugby [5].

SCJ dislocation can occur with two mechanisms. One occurs by applying a compressive force to the shoulder from the posterolateral side and the other occurs by a direct force to the clavicle from the anteromedial aspect [5,6]. In these traumas, at the first presentation, the patient may experience neck and chest pain as well as related shoulder pain caused by the trauma. Additionally, there may be accompanying complaints such as respiratory distress, dysphagia, and numbness in the upper extremities. Subsidence can also be where the clavicle articulates with the sternum. These give us preliminary information about the SCJ protruding backward [3,4].

This report describes the uncomplicated management of posterior dislocation of the SCJ with timely and correct intervention, non-surgical treatment, closed reduction maneuver, and rehabilitation steps.

## Case presentation

**Figure 1:**
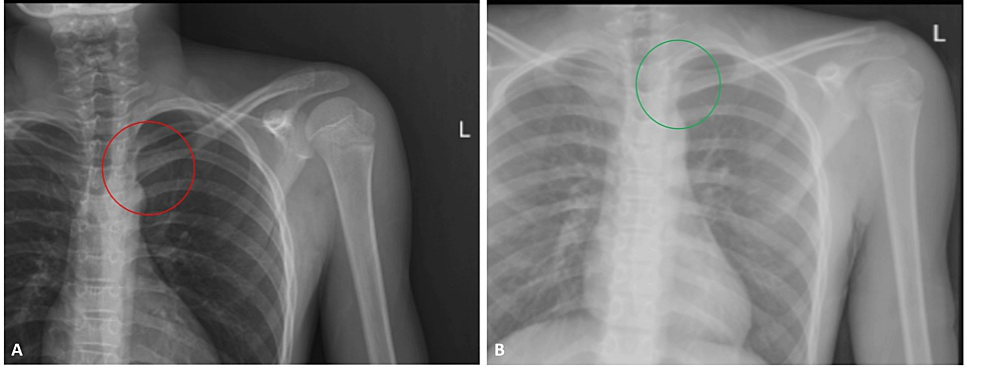
Left shoulder anteroposterior radiographs before the and after the reduction (A) Left shoulder anteroposterior radiograph before the reduction. The left sternoclavicular joint is marked with a red circle; (B) Left shoulder anteroposterior radiograph after the reduction. The left sternoclavicular joint is marked with a green circle.

A 13-year-old male patient presented to the emergency department with complaints of pain radiating from the left shoulder to the chest and neck, numbness in the left upper extremity, and difficulty in breathing after receiving a direct elbow strike to the left clavicle from the anteromedial aspect while playing football. On physical examination, the patient's left SCJ was sunken backward. We detected hypoesthesia in the left upper extremity compared to the other upper extremity. There was also motor weakness in the left upper extremity. The left shoulder passive joint range of motion was full. However, during movement, he had localized and severe pain on the left lateral aspect of the proximal sternum, and the complaint of numbness in the left upper extremity increased. The patient's oxygen saturation was 93-95%, and respiratory rate was between 20-25 breaths per minute. The patient had posteroanterior chest and anteroposterior shoulder radiographs done. There was no obvious dislocation on plain radiographs (Figure 1A). 

**Figure 2:**
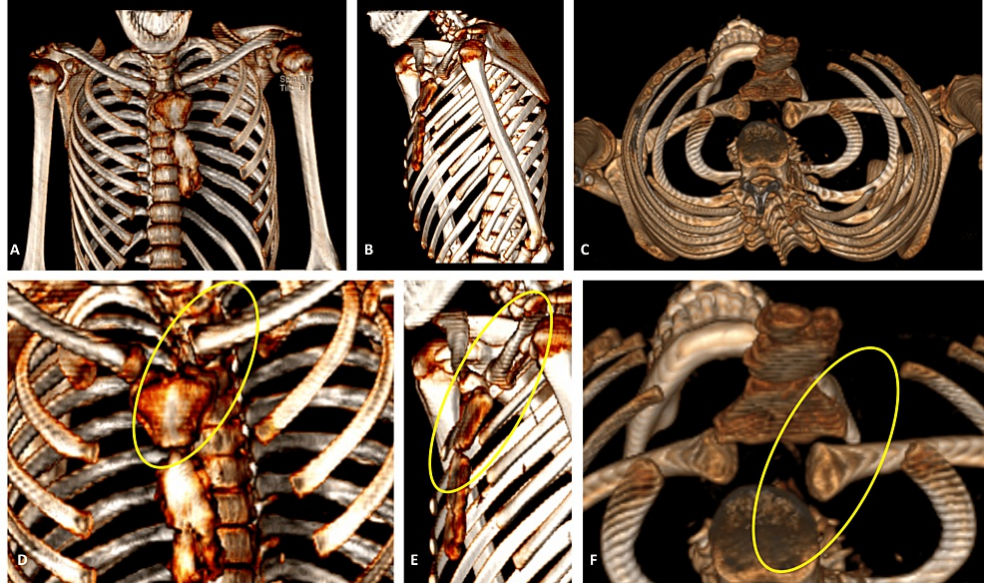
Three-dimensional reconstructed computed tomography of the patient after the trauma before the closed reduction Anterior (A), lateral (B), inferior (C), magnified anterior (D), magnified lateral (E), and magnified inferior (F) views. The left sternoclavicular joint is marked with a yellow ellipse.

**Figure 3:**
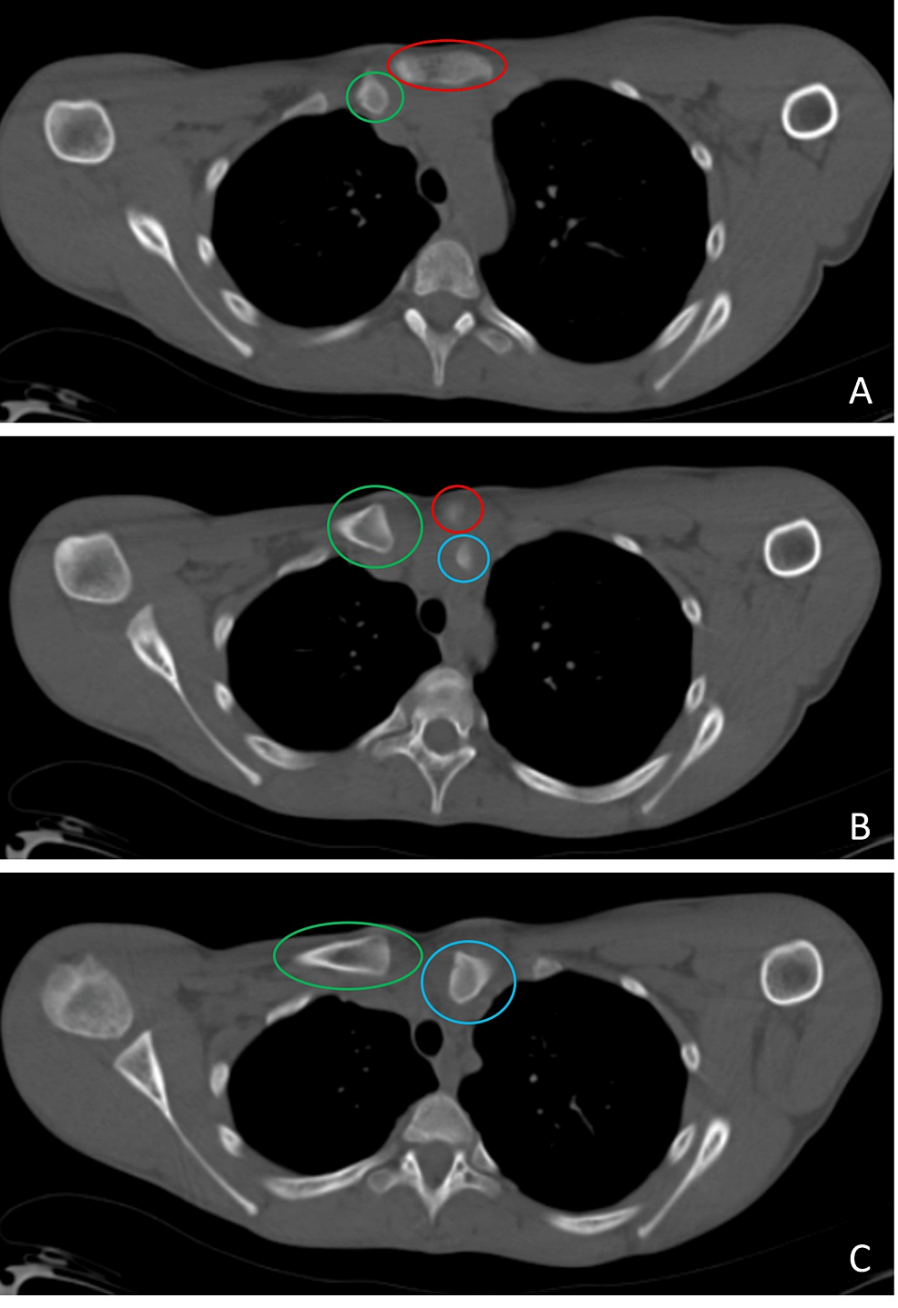
Axial computed tomography sections of the patient after the trauma before the closed reduction The left sternoclavicular joint posterior dislocation is remarkable with the consecutive axial computed tomography sections (A, B, C) after the trauma before the closed reduction. The proximal end of the left clavicle is marked with a blue circle. The proximal end of the right clavicle is marked with a green circle. The sternum is marked with a red ellipse and circle.

The preliminary diagnosis was posterior dislocation of the SCJ, and computed tomography with three-dimensional reconstruction was performed. This confirmed posterior dislocation of the left SCJ (Figure 2 and Figure 3). 

A closed reduction maneuver was performed under sedation in the operating room immediately within the first hour of presentation. We used the technique described by Honeycutt et al. [7] for the closed reduction. With the shoulder in 90 degrees abduction, we applied lateral traction and slowly extended the shoulder. After closed reduction was achieved, the patient was placed in a shoulder arm sling. No neurovascular complications were detected after the maneuver. A serendipity view with the fluoroscopy showed successful joint reduction with appropriate anatomical alignment. Postoperative radiographs also supported proper reduction (Figure 1B).

Ice application, non-steroid anti-inflammatory drug treatment, and monitoring for the neurovascular deficit were explained to the patient, who was checked weekly. During the follow-up, it was observed that the motor weakness and hypoesthesia in the left upper extremity improved. In the fourth week, the shoulder arm sling was removed and a serendipity radiograph was taken. After seeing that the joint was in place and the patient had no complaints, rehabilitation was started with shoulder pendulum exercises. In the sixth week, shoulder movements regained normal range. We started strengthening exercises and allowed the patient to bear weight on the left shoulder in the sixth week. In the eighth week of follow-up, the patient returned to sports with a full range of motion of the joints around the shoulder without any structural deformity or loss of strength.

## Discussion

SCJ dislocation is rare, and posterior dislocation of the SCJ is even rarer [1]. Posterior dislocation is vital due to the complications that may occur due to its proximity to the mediastinal structures, and it requires intervention within 48 hours for closed reduction to be highly effective [7]. In some sources, success has also been demonstrated in closed reductions performed for up to 10 days [8]. The rarity of this injury can be attributed to the tight joint capsule of the SCJ and the incomplete ossification of the medial clavicle until the age of 25 [2]. Thus, posterior dislocation of the SCJ under the age of 25 can be attributed to the fracture of the physis of the medial joint surface of the clavicle, and these injuries can be managed with a conservative treatment approach [9].

Posterior dislocation of the SCJ generally occurs by applying direct force to the clavicula from the anteromedial aspect [5,6]. In our patient, the dislocation was due to a direct elbow strike to the anteromedial aspect of his left clavicle while playing football, and the patient was admitted to the emergency department with complaints of chest, neck, and left shoulder pain and numbness in the left upper extremity. Even the symptoms show us that urgent intervention is required. This injury can be detected with anteroposterior and serendipity radiographs of the clavicle, but these radiographs are not always successful. Sometimes, a mild asymmetry seen on the radiograph can also be guiding [7]. In our case, the direct radiograph did not help much in the diagnosis. Therefore, we performed an advanced examination with computed tomography with three-dimensional reconstruction for the diagnosis. If there is any suspicion about this type of injury, further examination helps in the diagnosis. The fact that the patient was dyspneic and tachypneic after the trauma made us suspicious. We see the importance of anamnesis and physical examination too. Only the direct radiograph may not be enough for a correct diagnosis.

Serendipity radiography can be used for post-reduction control [10]. We controlled the reduction with a serendipity view with the fluoroscopy, and there was no asymmetry. At the same time, the subsidence in the SCJ was no more after the reduction. The patient's dyspnea also disappeared. After the reduction, we followed the patient with weekly checks by providing shoulder immobilization with a shoulder arm sling. Regardless of the reduction method, even if the joint is stable after treatment, fixing the shoulder with a shoulder arm sling for ligament healing and restricting shoulder movements, as it may delay healing, is necessary [11].

## Conclusions

Posterior dislocation of the SCJ is a rare orthopedic emergency that requires urgent intervention. Direct radiography can sometimes be incomplete or misleading. Therefore, in the presence of alarming symptoms and findings such as dyspnea, tachypnea, and neurovascular damage, rapid and accurate diagnosis can be achieved with the help of three-dimensional computed tomography. With 90 degrees of shoulder abduction under sedation, the closed reduction was completed by lateral traction and slight extension of the shoulder joint. Then, the shoulder joint was immobilized with a shoulder arm sling. The patient was followed up weekly with a shoulder arm sling without surgery. In the fourth week, pendulum exercises were started and in the sixth week, strengthening exercises with gradual load bearing. With timely intervention and rehabilitation, the patient could return to sports in the eighth week without any complaints, deformities, movement restrictions, or complications.
